# Bronchial arterial embolization for an unruptured bronchial artery aneurysm in patient with hemoptysis

**DOI:** 10.1007/s11739-024-03647-y

**Published:** 2024-05-21

**Authors:** Keisuke Suzuki, Ryozo Kai, Takaho Hirano, Masaharu Yagi, Kenji Dohi

**Affiliations:** 1 Department of Emergency, Critical and Disaster Medicine, Showa University School of Medicine, 1-5-8 Hatanodai, Shinagawa-ku, Tokyo, 142-8666 Japan; 2 Department of Radiology, Division of Radiology, Showa University School of Medicine, 1-5-8 Hatanodai, Shinagawa-ku, Tokyo, 142-8666 Japan; 3 Department of Radiological Technology, Showa University Hospital, 1-5-8 Hatanodai, Shinagawa-ku, Tokyo, 142-8666 Japan; 4 Graduate School of Health Science, Showa University, 1865 Tokaichibacho, Midori-ku, , Yokohama-shi, Kanagawa 222-8555 Japan

A 65-year-old man with a history of tuberculosis (TB) was transferred to our emergency department with hemoptysis. Oxygen saturation was decreased on arrival (SpO2, 85% on room air). Laboratory tests revealed a hemoglobin level of 7.7 g/dL (reference range 14.0–18.0 g/dL) and D-dimer of 1.9 μg/mL (reference range 0–1.0 μg/mL). Contrast-enhanced computed tomography (CECT) revealed bilateral dilatation of the bronchial and right intercostal arteries, and a right bronchial artery aneurysm (BAA: 20 mm × 16 mm × 27 mm) (Fig. 1a, b). As the BAA had not ruptured and the hemoptysis resolved, the patient was admitted to the intensive care unit (ICU) for monitoring with conservative treatment. However, due to continued production of large amounts of bloody sputum, micro-coaxial catheter bronchial arterial embolization (BAE) was attempted on day five under local anesthesia and intravenous sedation. Angiography revealed that the bronchial artery aneurysm was located immediately after the aortic bifurcation (Fig. 1c, d). Due to its location, embolization of the aneurysm was not possible intraoperatively. Therefore, per the usual BAE policy, a gelatin sponge was administered distally to the aneurysm to embolize the dilated bronchial and intercostal arteries. The hemoptysis gradually decreased, and the patient was discharged from the ICU on day 17. Follow-up CECT revealed aneurysm thrombosis.

**Fig. 1:**
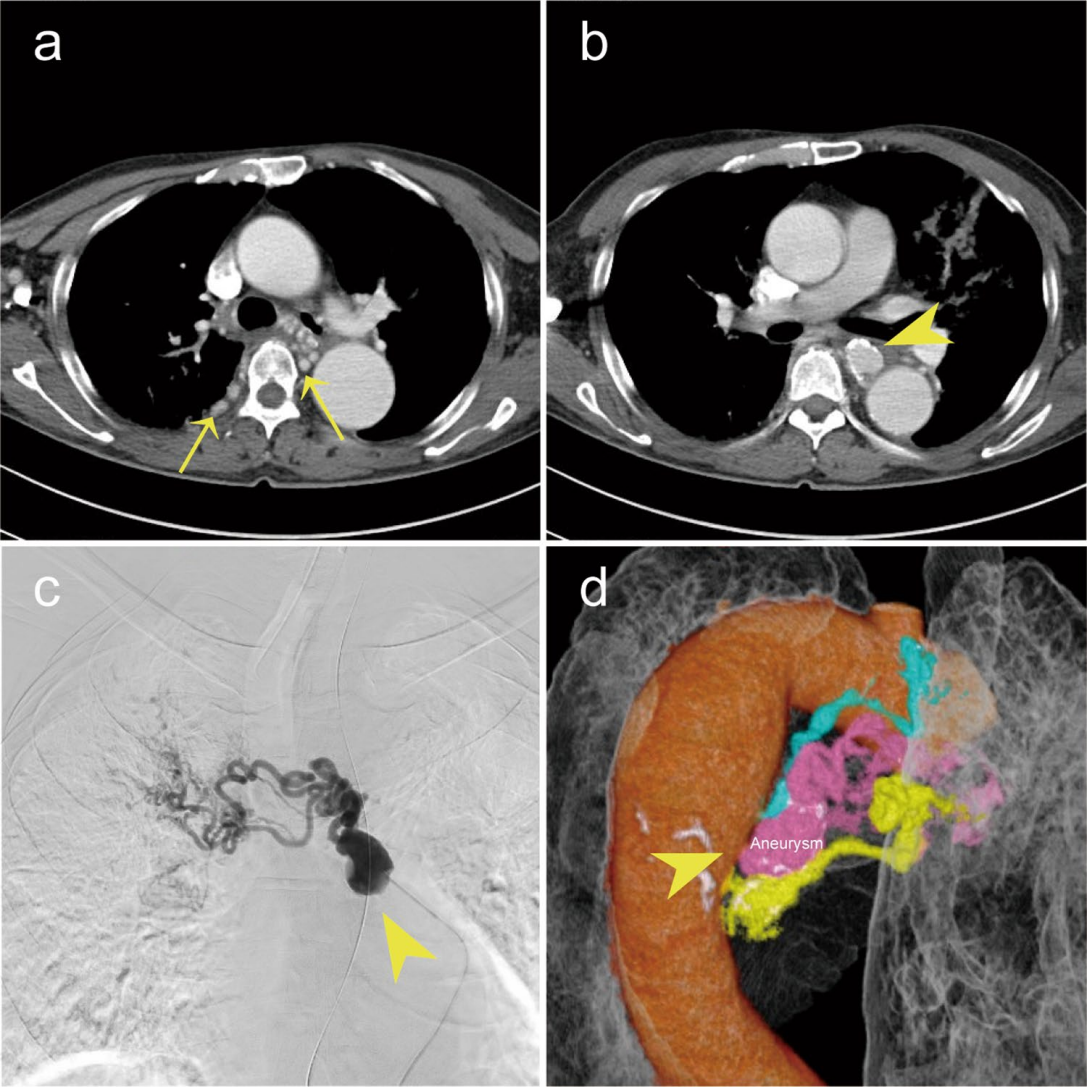
**a** Axial Contrast-enhanced CT of dilated bronchial and intercostal arteries (arrow). **b** Axial Contrast-enhanced CT of bronchial artery aneurysm (arrowhead). **c** Digital subtraction angiography of bronchial artery aneurysm (arrowhead) and dilated bronchial artery. **d** Contrast-enhanced CT image reconstructed in 3D and pseudo-colored. View from the back right of the patient, demonstrates no bronchial artery aneurysm (arrowhead)

BAA is a rare disease, with an incidence of less than 1% as detected on bronchial angiography [1]. It is most commonly associated with bronchiectasis and possible causes include hypertension, chronic obstructive pulmonary disease, vasculitis, chronic bronchopulmonary infection, TB, and trauma [2]. In this case, other diseases were ruled out and chronic inflammation due to TB was considered the cause. Hemoptysis is caused by vascularization of the lung parenchyma by bronchial and non-bronchial systemic arteries. These include the intercostal, inferior thoracic, internal mammary, and collateral arteries from the intercostal and subclavian arteries [3]. In this case, the BAA was not considered a direct cause of hemoptysis, because the aneurysm had not ruptured and was located in the posterior mediastinum, not in the lung field. Dilated bronchial and intercostal arteries are considered to be the cause of hemoptysis. Treatment can be either open thoracotomy or endovascular therapy; however, trans arterial embolization is considered a good first choice for the treatment of BAA, given its minimally invasive nature. Coil embolization is difficult when the neck of the aneurysm is inadequate, as in this case. In such cases, a thoracic stent graft may be used to cover the BAA [4]. However, immediate performance of this procedure is difficult in emergency settings. In this case, the patient did not experience rupture; therefore, we only embolized the peripheral dilated bronchial artery beyond the aneurysm. The symptoms improved and the aneurysm thrombosed, which was sufficient for temporary hemostasis. Adverse effects of BAE include bronchial necrosis and recurrence of hemoptysis. The recurrence-free rate of gelatin sponges after one year of embolization is as low as 45% [5]. Careful follow-up is required, and embolization of the BAA itself should be considered in the event of re-bleeding.

## Data Availability

All relevant data pertaining to this case are contained in the article.
